# VE-cadherin cleavage by ovarian cancer microparticles induces β-catenin phosphorylation in endothelial cells

**DOI:** 10.18632/oncotarget.6677

**Published:** 2015-12-19

**Authors:** Hamda Al Thawadi, Nadine Abu-Kaoud, Haleema Al Farsi, Jessica Hoarau-Véchot, Shahin Rafii, Arash Rafii, Jennifer Pasquier

**Affiliations:** ^1^ Qatar Research Leadership Program, Qatar Foundation, Doha, Qatar; ^2^ Stem Cell and Microenvironment Laboratory, Weill Cornell Medical College in Qatar, Education City, Qatar Foundation, Doha, Qatar; ^3^ Department of Genetic Medicine, Weill Cornell Medical College, NY, USA

**Keywords:** microparticles, ovarian cancer, tumor microenvironment, β-catenin, angiogenesis

## Abstract

Microparticles (MPs) are increasingly recognized as important mediators of cell-cell communication in tumour growth and metastasis by facilitating angiogenesis-related processes. While the effects of the MPs on recipient cells are usually well described in the literature, the leading process remains unclear. Here we isolated MPs from ovarian cancer cells and investigated their effect on endothelial cells. First, we demonstrated that ovarian cancer MPs trigger β-catenin activation in endothelial cells, inducing the upregulation of Wnt/β-catenin target genes and an increase of angiogenic properties. We showed that this MPs mediated activation of β-catenin in ECs was Wnt/Frizzled independent; but dependent on VE-cadherin localization disruption, αVβ3 integrin activation and MMP activity. Finally, we revealed that Rac1 and AKT were responsible for β-catenin phosphorylation and translocation to the nucleus. Overall, our results indicate that MPs released from cancer cells could play a major role in neo-angiogenesis through activation of beta catenin pathway in endothelial cells.

## INTRODUCTION

Clinical and experimental evidences indicate that tumor initiation and progression is intimately related to the complex dialogue between tumor microenvironment and malignant cells [1, 2]. In our previous study we demonstrated that ovarian and breast cancer cell microparticles promoted activation of endothelial cells (ECs) through Akt phosphorylation and Arf6 up-regulation [3]. MPs are submicron vesicles ranging from 200 nm to 1um in diameter, shed from the plasma membrane of a variety of cells and released into the extracellular microenvironment [4]. MPs act as messengers delivering variety of cargos, such as cell surface receptors, multidrug resistance proteins, cytokines, proteins and mRNA to distal cells [5-7]. Once released from the donor cells, MPs travel through extracellular milieu to the plasma membrane of the recipient cells [8, 9], activating target cell receptors [10, 11] or mediating signaling directly by transferring their content within target cells [12].

Cancer cell derived MPs are involved in angiogenesis promotion. For instance, glioblastoma (GBM) cell-derived MPs are efficiently internalized by endothelial cell human umbilical vein (HUVEC) and primary human brain microvascular cells (HBMECs), inducing microvascular sprouting [13]. Renal cancer cells derived MPs promote angiogenesis via up regulation of VEGF expression in HUVECs [14]. In colorectal cancer, Yoon et al. demonstrated that MPs promote endothelial cell migration via ERK1/2 and JNK signaling pathways and lipid raft-mediated endocytosis [15]. MPs derived from colon cancer cells transfer ΔNp73 to HUVECs resulting in increased proliferation [16]. In nasopharyngeal carcinoma, MPs increased tubulogenesis, migration and invasion of HUVECs in a dose-dependent manner [17]. All reports concordantly describe an increase of the angiogenic phenotype upon exposure of ECs to cancer derived MPs. The precise mechanism or pathway leading to activation of ECs is not clearly described and might be specific to tumor type, and play a role in resistance to anti-angiogenic treatment. The β-catenin pathway regulates a variety of cellular functions such as proliferation, polarity, adhesion, differentiation and hemostasis [18]. In angiogenic processes during embryonic development, β-catenin signaling plays a crucial role in balancing Notch-induced cell cycle arrest promoting vascular stability [19]. A baseline level of Wnt/β-catenin activity can be detected during vascular development and tumor angiogenesis, suggesting a mechanism where the level of Wnt/β-catenin is tightly balanced and crucial for proper vasculature development [19-22]. High activation of β-catenin transcriptional signaling could be detected through embryonic development in different types of vessels such as retina or brain microvasculature [23–25].

Angiogenesis is crucial in advanced ovarian cancer where anti-angiogenic treatment targeting VEGF demonstrated an effect on progression free survival [26, 27]. However, despite initial sensitivity to chemotherapy and anti-angiogenic, most patients will display a recurrence within the abdominal cavity associated to resistance to anti-angiogenic therapies and VEGF independent activation of ECs [24].

Here, we investigated the effect of MPs derived from ovarian cancer cell lines (OCC-MPs) on ECs. We demonstrated that ovarian cancer MPs trigger β-catenin activation in ECs increasing angiogenic properties. We showed that the activation of β-catenin in ECs by OCC-MPs was Wnt/Frizzled independent; but related to integrin and subsequent MMP activation leading to VE-cadherin disruption from the membrane. Finally we established that Rac1 and AKT were the intracellular mediators of β-catenin phosphorylation and translocation to the nucleus.

## RESULTS

### Ovarian cancer MPs are able to trigger β-catenin activation in endothelial cells

**Figure 1:**
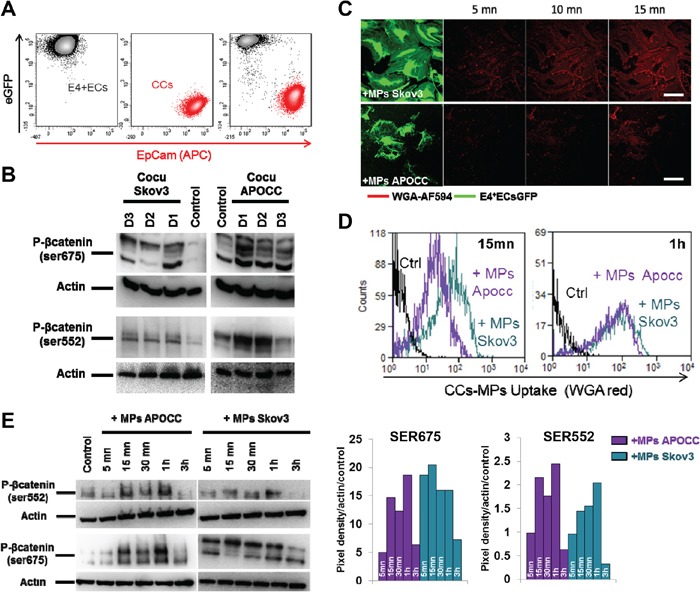
Ovarian cancer cells MPs trigger β-catenin phosphorylation in endothelial cells **A.** Flow cytometry cell sorting chart. eGFP-E4+ECs (black) and APOCC or Skov3 (red) were gated through eGFP fluorescence intensity and an APC conjugated EpCam staining. eGFP-E4+ECs were sorted as eGFP^+^/EpCam^−^. **B.** E4+ECs after co-culture with OCC. Phosphorylation of β-catenin at the site Ser 675 and Ser 552 were quantified by western blot. E4+ECs display a phosphorylation of β-catenin for the sites studied after co-culture with Skov3 or APOCC. **C–D.** OCC-MPs uptake by E4+ECs. MPs from APOCC or Skov3 were extracted from 80% confluent cells and labelled with Alexa Fluor 594 conjugated-wheat germ agglutinin (WGA). MPs uptake by eGFP-E4+ECs was quantified by confocal microscopy (screenshot from time-lapse recording, C) or flow cytometry (D). E4+ECs are able to uptake Skov3-MPs or APOCC-MPs in less than 15 minutes. *Scale bar: 10 μm.* **E.** Phosphorylation of β-catenin. E4+ECs, serum-starved for 24 h, were treated with MPs extracted from APOCC (left panel) or Skov3 (right panel). Western blots for the phosphorylation of β-catenin at sites Ser675 and Ser552 were performed. The bar graphs represent the pixel density of each band normalized using actin band and the control of the experiment. OCC-MPs phosphorylate β-catenin at Ser675 and Ser552 in E4+ECs.

To investigate β-catenin activation, we established a co-culture of eGFP-E4+ECs, with two ovarian cancer cell lines Skov3 and APOCC (ovarian cancer cell line derived in our laboratory from ascites of a patient with Stage III serous adenocarcinoma [3]). Upon 24h co-culture, western blot analysis of sorted ECs illustrated β-catenin phosphorylation at 2 different sites (Ser 675 and Ser 552) (Figure 1A–1B). The use of transwell chambers (0.4 μm) didn't abrogate this activation suggesting the implication of secreted molecules or released vesicles like MPs (Supplementary Figure 1). To track spontaneous excretion of MPs, we stained OCCs using WGA (wheat germ agglutinin). For all different cell lines, culture supernatants yielded pellets consisting of a similar population of vesicles <1 μm in diameter (FACS or Confocal, Supplementary Figure 2). Using confocal microscopy analysis and flow cytometry, we showed the uptake of OCC-MPs by E4+ECs. (Figure 1C–1D). The uptake of Skov3-MPs by the ECs was higher compared to APOCC-MPs at earlier time points (Figure 1D).

We then investigated the role of OCC-MPs on β-catenin activation, and we showed that it could reproduce the phosphorylation of Ser522 and Ser675 obtained with co-cultures after 15 min and up to 1h of incubation of ECs with OCC-MPs (Figure 1E).

### Ovarian cancer cells MPs induce β-catenin translocation to the nucleus in ECs and trigger angiogenic properties

**Figure 2:**
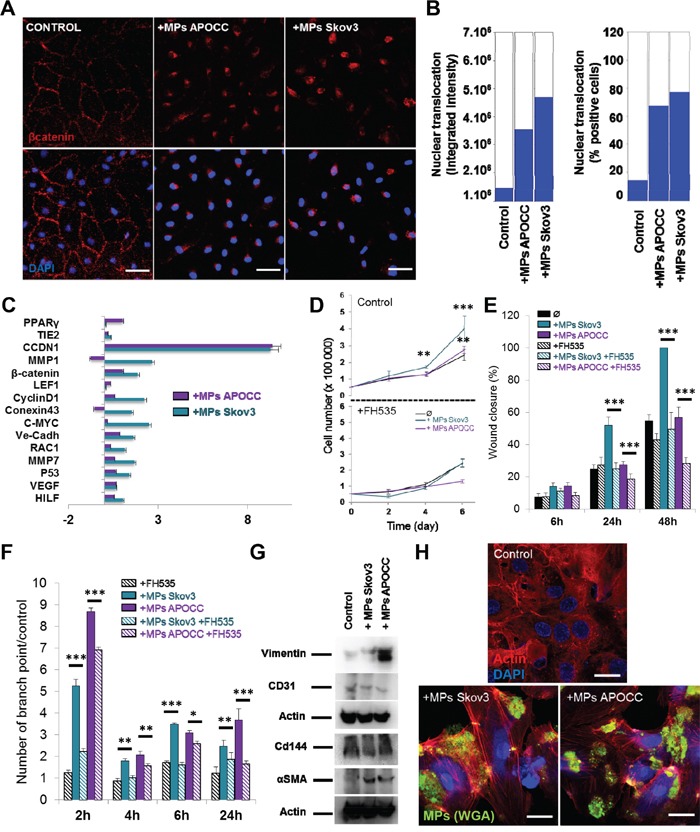
Ovarian Cancer cells MPs induce β-catenin translocation to the nucleus in ECs and trigger angiogenesis properties **A.** Localization of β-catenin. E4+ECs treated or not (control) with MPs from Skov3 or APOCC for one hour were stained for β-catenin and analyzed by High-Content analysis microscopy. In the control condition, β-catenin is localized mainly at the membrane of the cells. After incubation with OCC-MPs, β-catenin could be found only in the nucleus of the E4+ECs. *Scale bar: 20 μm.* **B.** Quantification of β-catenin translocation. The experiments presented in A were quantified with ImageXpress Micro XLS Widefield High-Content Analysis System using 5 wells with 50 images taken by well. The bar graphs represent the fluorescence intensity of the staining inside the nucleus (left panel) or the percentage of positive cells for staining in the nucleus (right panel). **C.** Real-time qPCR. The relative quantification of genes under control of β-catenin pathway was performed by real-time qPCR on E4+ECs after treatment with MPs from APOCC (purple) or Skov3 (green). Most of β-catenin downstream genes were upregulated compared to control. Relative transcript levels are represented as the log10 of ratios between the 2 subpopulations of their 2^−ΔΔCp^ real-time PCR values. **D.** Proliferation assay. E4+ECs were plated and counted every 2 days in presence or not of MPs from OCC (Control, top panel). The same experiment was repeated in presence of 10 μM of FH535, an inhibitor of β-catenin pathway. MPs from Skov3 (green) or APOCC (purple) did not increase the proliferation of E4+ECs when β-catenin is inhibited. **E.** Wound closure assay. Migration of E4+ECs with or without FH535 (10 μM) was evaluated in the presence or absence of OCC-MPs. Motility of E4+ECs was reduced by β-catenin inhibition. **F.** Tube formation assay. E4+ECs with or without FH535 (10 μM) were plated on matrigel layer in presence or not of OCC-MPs. MPs from Skov3 and APOCC were able to increase the number of tubes and their stability only when β-catenin was not inhibited. **G.** Evaluation of mesenchymal and endothelial markers in E4+ECs. E4+ECs were treated with MPs from Skov3 or APOCC every 2 days during 6 days. Western blot for mesenchymal (Vimentin and αSMA) and endothelial (CD31 and CD144) markers were performed. While endothelial markers were conserved, mesenchymal markers were expressed in E4+ECs after incubation with OCC-MPs. **H.** F-actin polymerization in E4+ECs after treatment with OCC-MPs. E4+ECs were grown on glass bottom slides and actin cytoskeleton was revealed by a phalloïdin-fluorescein (1 μg/mL) labeling. Fluorescence microscope series of adherent E4+ECs unstimulated or stimulated with OCC-MPs and stained with Alexafluor488. OCC-MPs induced stress fibers in E4+ECs. *Scale bar: 20μm*. p < 0.05 (*), p < 0.01 (**) or p < 0.001 (***).

The activation of β-catenin is associated with its phosphorylation at different sites and its translocation from membrane bound complexes (cadherin) to the nucleus. Therefore, we investigated whether OCC-MPs uptake by ECs affected β-catenin localization in E4+ECs. β-catenin translocation to the nucleus was observed in E4+ECs after 1h of incubation with OCC-MPs (Figure 2A–2B). We confirmed the effect of β-catenin translocation with the up-regulation of target genes using a β-catenin q-PCR array after an incubation of 12h with CC-MPs displaying increase in target genes such as LEF1 (Fold 2.25 and 2.19 for Skov3 and APOCC respectively), VEGF (Fold 4.69 and 4.31 for Skov3 and APOCC respectively) or Rac1 (Fold 13.71 and 2.07for Skov3 and APOCC respectively; Figure 2C). OCC-MPs treatment increased ECs proliferation (Figure 2D), migration (Figure 2E) and tube formation (Figure 2F) concordant with endothelial β-catenin activation. These functional effects were all abolished by using β-Catenin inhibitor FH535. ECs are known to be plastic and acquire mesenchymal traits participating to tumor promotion [28, 29]. We noticed that long term OCC-MPs treatment (6 days) and β-catenin activation induced mesenchymal traits in ECs (Vimentin and α-sma expression, increased stress fibers) while maintaining the endothelial trait (CD144 and Cd31; Figure 2G-2H).

### Ovarian cancer cells MPs uptake by ECs is dependent on integrin activation

**Figure 3:**
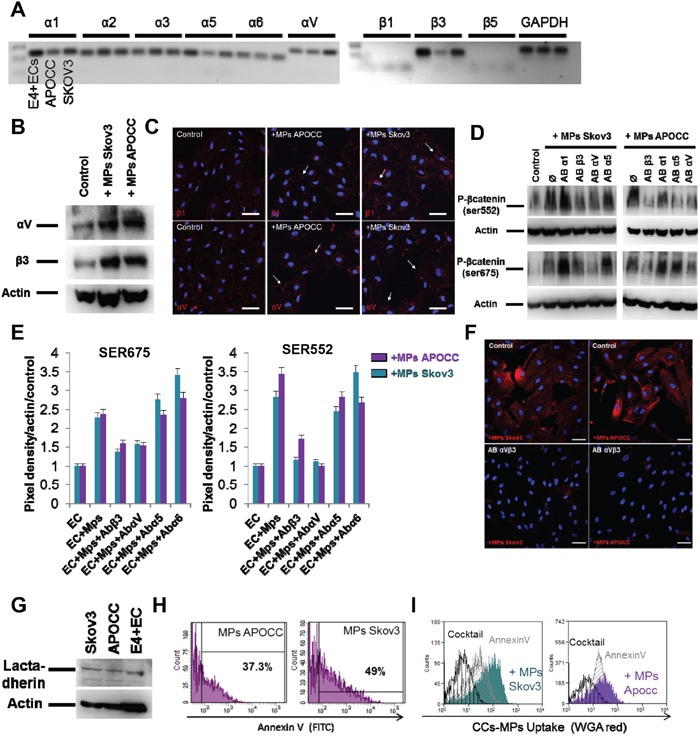
Ovarian Cancer cells MPs uptake by ECs is dependent on integrin activation **A.** Integrin expression in E4+ECs, APOCC and Skov3. PCR for integrins α1, 2, 3, 5, V and β1, 3, 4 were performed. Integrins β3, α1, 5 and V were the main integrins expressed. **B.** Integrin expression. E4+ECs, serum-starved for 24 h, were treated with OCC-MPs during 15 minutes. Western blots for the integrins β3 and αV were performed. OCC-MPs were able to increase the expression of the two studied integrin in the E4+ECs. **C.** αV and β3 integrins localization. E4+ECs, serum-starved for 24 h, were treated or not (control) with MPs from Skov3 or APOCC for one hour. The staining for integrin β1 (top panel) or αV (bottom panel) displayed integrins clustering upon OCC-MPs treatment (arrows). *Scale bar: 20 μm.* **D.** β-catenin phosphorylation in the presence of integrin inhibitors. E4+ECs, serum-starved for 24 h, were treated or not with a monoclonal antibody against integrins α1, α5, αV or β3 prior to the incubation with OCC-MPs during 15 minutes. Western blots for the phosphorylation of β-catenin at sites Ser675 and Ser552 were performed. The blockade of integrins αV and β3 inhibited the phosphorylation of β-catenin induced by OCC-MPs. **E.** Quantification of β-catenin phosphorylation under integrins blockade. The bar graphs represent the pixel density of each band normalized using actin band and the control of the experiment. **F.** OCC-MPs uptake by E4+ECs in presence of integrin αVβ3 monoclonal blocking antibody. MPs from APOCC or Skov3 were extracted from 80% confluent cells and labelled with Alexa Fluor 594 conjugated-wheat germ agglutinin (WGA). MPs uptake by eGFP-E4+ECs after 1 h was quantified by confocal microscopy. E4+ECs were not able to uptake Skov3-MPs or APOCC-MPs in presence of integrin inhibitors. *Scale bar: 10 μm.* **G** Lactadherin expression. E4+ECs, Skov3 and APOCC were analysed in western blots for the Lactadherin. The inhibition of integrins αV and β3 were able to inhibit the phosphorylation of β-catenin induced by OCC-MPs. **H.** Quantification of phosphatidyl serine in OCC-MPs. MPs extracted from Skov3 or APOCC were stained with FITC-annexin V. Quantification of positive MPs were performed by flow cytometry. A large number of OCC-MPs were positive for annexin V staining revealing the presence of phosphatidyl serine at the surface of MPs. **I.** OCC-MPs uptake by E4+ECs. MPs from APOCC or Skov3 were extracted from 80% confluent cells and labelled with Alexa Fluor 594 conjugated-wheat germ agglutinin (WGA). E4+ECs control (plain plot) or pre-treated with annexin V (grey plots) or with a cocktail containing annexin V and an antibody against lactadehrin (black plot) were exposed to the stained OCC-MPs for 1 h. The quantification was performed by flow cytometry. MPs uptake by E4+ECs was inhibited completely by the combination of annexin V and the antibody against lactadherin.

We first characterized the integrin repertoire of Skov3, APOCC and E4+ECs (Figure 3A). Among the different integrins expressed by ECs we selected 3 α-subunits (1, 5, and V) and 1 β-subunit (β3) as their crucial role in angiogenesis and tumor progression has been well documented, and that treatment of ECs by OCC-MPs increased its expression and induced clustering and accumulation of αVβ3 integrin at the border of E4+EC (Figure 3B, 3C). Using blocking antibodies against each integrin, we demonstrated a role of integrins αVβ3 in the phosphorylation of Ser522 and Ser675 of β-catenin when treated with OCC-MPs (Figure 3D–3E).

Integrins are involved in MPs uptake through phosphatidyl serine (PS) interaction with lactadherin [30]. Blocking antibodies against αVβ3, abolished the uptake of OCC-MPs (Figure 3F). We confirmed the presence of lactadherin in our cell lines (Figure 3G) and PS in the OCC-MPs displaying positive annexin V staining (Figure 3H). Using annexin V or a cocktail with annexin V and a blocking antibody against lactadherin, we demonstrated the inhibition of OCC-MPs uptake by ECs when the interaction between lactadherin and PS was disrupted (Figure 3I). Altogether, we demonstrated that OCC-MPs uptake required αVβ3 activation and lactadherin and PS interaction. This uptake resulted in β-catenin activation.

### β-catenin activation in ECs is dependent on MMP induced VE-cadherin disruption

**Figure 4:**
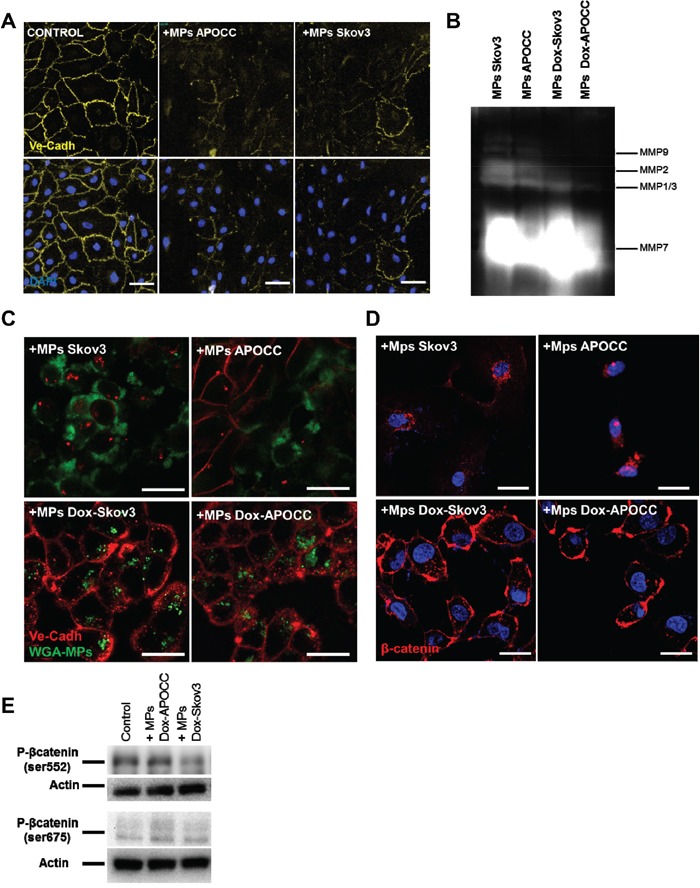
β-catenin activation in ECs is dependent on VE-cadherin localization disruption mediated by MMPs activity **A.** Localization of VE-cadherin. E4+ECs treated or not (control) with MPs from Skov3 or APOCC for one hour were strained for VE-cadherin and analyzed by High-Content analysis microscopy. In the control condition, VE-cadherin is localized at the membrane of the cells. After incubation with OCC-MPs, VE-cadherin disappears from E4+ECs cell junctions *Scale bar: 20 μm.* **B.** Matrix metalloproteases activity. APOCC and Skov3 were treated with doxycycline (1μg/ml) for 24h (Dox-Skov3 and Dox-APOCC). MPs were extracted from 80% confluent cell culture of Skov3, APOCC, Dox-Skov3 and Dox-APOCC. A zymogram was performed to assess the matrix metalloproteases (MMP) activity in OCC-MPs. Both Skov3 and APOCC demonstrated a strong MMPs activity, while the treatment with doxycline significantly reduced the metalloprotease activity. **C.** VE-cadherin localization. MPs extracted from Skov3, APOCC, Dox-Skov3 and Dox-APOCC were labelled with Alexa Fluor 488 conjugated-wheat germ agglutinin (WGA). E4+ECs were treated for 1h with the MPs and a staining for VE-cadherin was performed. Confocal images revealed that even if the Dox-OCC-MPs were uptaken by E4+ECs, VE-cadherin was not disrupted from the membrane. *Scale bar: 20μm.* **D.** β-catenin localization. E4+ECs were treated for 1h with the MPs extracted from Skov3, APOCC or from the same cells pre-treated for 24 with 1μg/ml of doxycycline (Dox-Skov3 and Dox-APOCC). Staining for β-catenin was performed and localization of β-catenin was studied by confocal microscopy. When E4+ECs were treated with OCC-MPs, β-catenin was translocated to the nucleus. While Dox-OCC-MPs, failed to induce β-catenin translocation. *Scale bar: 10 μm.* **E.** Phosphorylation of β-catenin. E4+ECs, serum-starved for 24 h, were treated with MPs extracted from Dox-APOCC or Dox-Skov3 for 15 minutes. Western blots for the phosphorylation of β-catenin Ser675 and Ser552 were performed. Dox-OCC-MPs were not able to phosphorylate β-catenin at the two sites.

In its inactive form, β-catenin is located at the cytoplasmic side of the cell membrane as an element of cadherin-complexes [31]. In endothelial cell junctions, β-catenin is usually associated with VE-cadherin's cytoplasmic domain [32]. Hence, we investigated the effect of OCC-MPS on VE-cadherin. After 1h of incubation with OCC-MPs, VE-cadherin disappeared from ECs junction (Figure 4A). Matrix metalloproteases (MMPs) are involved in VE-cadherin disruption [33–35]. Using gelatin zymography, we showed MMPs activity in OCC-MPs (Figure 4B). To assess the role of MMPs in VE-cadherin disruption and β-catenin release, Skov3 and APOCC were treated with the MMP inhibitor doxycycline for 24h and MPs were then extracted. The gelatin zymography confirmed a nearly complete inhibition of MMPs 2 and 9 activities in OCC-MPs after treatment with doxycycline (Figure 4B). Inhibition of MMPs 2 and 9 activities abrogated VE-cadherin disruption but not OCC-MPs uptake (Figure 4C). After 1h of incubation with doxycycline treated OCC-MPs, β-catenin was still found localized at the cytoplasmic part of the membrane (Figure 4D), and its phosphorylation was abolished (Figure 4E). MMPs activity within OCCs-MPs induced VE-cadherin disruption and β-catenin translocation to the cytoplasm and its subsequent phosphorylation.

### Ovarian cancer cells MPs mediated phosphorylation of β-catenin in ECs depends on Rac1 and AKT but not on Wnt/Frizzled pathway

β-catenin can be activated through different cascades [36]. Wnt signaling represents one of the key molecular pathways regulating β-catenin activation. In the presence of Wnts, a conformational change of Frizzled and LRP5/6 leads to inactivation of β-catenin destruction complex, and accumulation of β-catenin in the cytoplasm [37]. β-catenin is not increased in ECs after incubation with OCC-MPs (Supplementary Figure 3A). Concordantly, no change could be observed at the phosphorylation sites Ser33-37 or Thr41/Ser45 which are involved in the destruction of β-catenin when frizzled is not activated (Supplementary Figure 3B). In our model of ECs, only Frizzled 1, 2, 4, 6, 7, 8 and LRP5 were expressed (Supplementary Figure 3C). Using blocking monoclonal antibodies against each expressed Fzd and LRP5, we showed no changes in inhibition of β-catenin phosphorylation at Ser552 and Ser675 (Supplementary Figure 3D), nor its translocation to the nucleus (Supplementary Figure 3E). Altogether our data suggest that OCC-MPs mediated phosphorylation of β-catenin in E4+ECs is Wnt/Frizzled independent.

**Figure 5:**
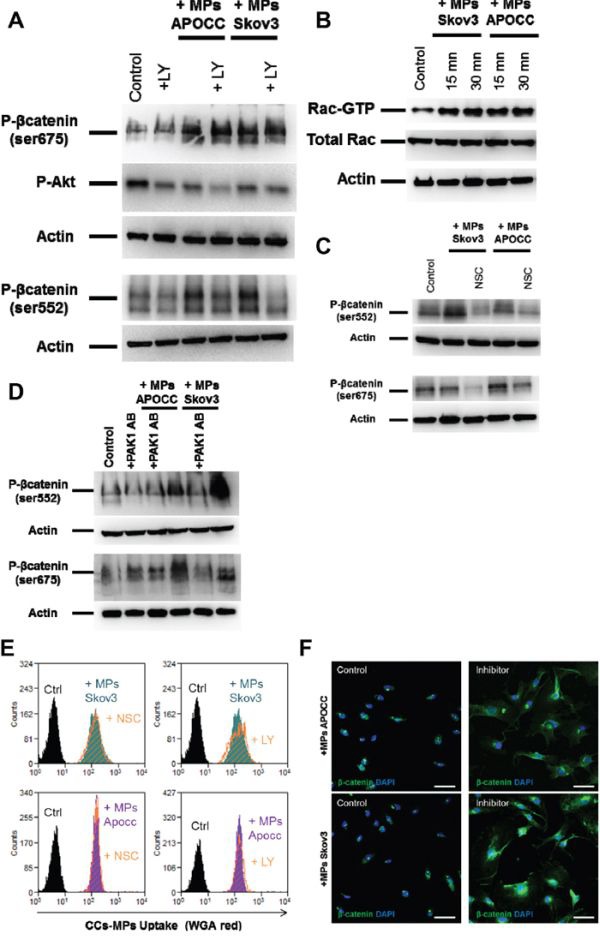
Rac1 and AKT are responsible for phosphorylation of β-catenin in ECs **A.** Phosphorylation of β-catenin in presence of Akt inhibitor. E4+ECs, serum-starved for 24 h, were pre-treated or not with LY 294002 (10μM). Phosphorylation of β-catenin Ser675 and Ser552 and of Akt at Ser473 were analyzed by western blot. While LY 294002 was able to inhibit the phosphorylation of Akt, it was only able to reduce the phosphorylation of β-catenin Ser552 but not Ser675. **B.** Rac1 activation. E4+ECs, serum-starved for 24 h, were treated with OCC-MPs for 15 or 30 minutes. Western blot analysis revealed an increase of Rac1 after incubation with OCC-MPs. **C.** Phosphorylation of β-catenin in the presence of Rac1 inhibitor. E4+ECs, serum-starved for 24 h, were pre-treated or not with NSC23766 (10μM). Phosphorylation of β-catenin Ser675 and Ser552 were analyzed by western blot. NSC23766 was able to reduce the phosphorylation of β-catenin at Ser552 and at Ser675. **D.** Phosphorylation of β-catenin in the presence of PAK1 blocking antibody. E4+ECs, serum-starved for 24 h, were pre-treated or not with a blocking antibody for PAK1. Phosphorylation of β-catenin Ser675 and Ser552 were analyzed by western blot. The inhibition of PAK1 reduced the phosphorylation of β-catenin Ser552 and Ser675. **E.** OCC-MPs uptake by E4+ECs in presence of inhibitor. MPs from APOCC or Skov3 were extracted from 80% confluent cells and labelled with Alexa Fluor 594 conjugated-wheat germ agglutinin (WGA). MPs uptake by eGFP-E4+ECs was quantified by flow cytometry in absence (purple and green plot) or presence of LY294002 or NSC23766 (orange plot). The inhibitors of AKT and Rac1 were not able to modify OCC-MPs uptake by E4+ECs. **F.** β-catenin localization. E4+ECs pre-treated or not with LY294002 and NSC23766, were treated for 1h with the MPs extracted from Skov3, APOCC. Staining for β-catenin was performed and localization of β-catenin was studied by confocal microscopy. When E4+ECs were pre-treated with LY294002 and NSC23766, β-catenin was not translocated to the nucleus but accumulated in the cytoplasm. *Scale bar: 10 μm.*

β-catenin Ser552 and 675 sites can be phosphorylated by PKA (protein kinase A), Akt or PAK1/Rac1 [38–40]. We first ruled out the implication of PKA in the phosphorylation of the 2 sites of β-catenin using the inhibitor H89 (Supplementary Figure 4). When ECs were treated with the Akt inhibitor, LY 294002 (10μM) prior to the incubation with OCC-MPs, the phosphorylation on Ser522 site was abolished but not the phosphorylation of Ser675 (Figure 5A). Then, we demonstrated that Rac1 was activated upon OCC-MPs treatment (Figure 5B). The inhibition of Rac1 activation by NSC23766, prevented the phosphorylation of β-catenin at the 2 sites (Figure 5C). PAK1 (Serine/threonine-protein kinase PAK 1) serves as a target for Rac1 and mediates Rac1 signaling. Using a monoclonal antibody against PAK1, we determined that the phosphorylation of β-catenin by Rac1 was mediated through PAK1 (Figure 5D). To demonstrate that Rac1 and Akt are involved only in the phosphorylation of β-catenin and not in the OCC-MPs uptake, we treated ECs with NSC23766 and LY294002 prior to incubation with OCC-MPs stained with WGA (Figure 5E). Both inhibitors did not decrease OCC-MPs uptake by E4+ECs. Nevertheless, the two inhibitors abolished the translocation of β-catenin to the nucleus (Figure 5F).

## DISCUSSION

We demonstrated the endothelial cell activation of β-catenin pathway by microparticles derived from ovarian cancer cells. Thus, revealing that integrins αVβ3 play a crucial role in MPs uptake and that MMPs activity leads to VE-Cadherin disruption and the release of β-catenin. Finally within the cytoplasm, Rac1 and Akt could mediate β-catenin phosphorylation and its translocation to the nucleus.

While Wnt signaling represents the major regulator of β-catenin, it is now well accepted that β-catenin is not just an element of Wnt cascade [36]. Over the past decades many groups have observed how specific binding partners can impact the nuclear-cytoplasmic distribution of β-catenin. For instance, VE-cadherin sequesters β-catenin to adherents’ junctions in ECs [41]. β-catenin together with VE-cadherin mobility both participate in endothelial cell-cell junction stabilization [42]. Recent studies emphasize the significant role of the β-catenin signaling pathway in physiological and pathological angiogenesis [24, 43]. Here, we demonstrated that the activation of β-catenin in ECs by OCC-MPs induced proliferative and mobility advantage. Moreover we demonstrated the increase of mesenchymal markers in ECs after exposure to OCC-MPs, illustrating the change in ECs phenotype. ECs are known to display a high level of plasticity depending on the context, a feature indispensable for tumor development [44, 45]. For instance, Bissel's group had described the ability of tumor-associated endothelium to change phenotype and function by processing signals received from their microenvironment [46–48]. In tumor context, cell-cell signaling induces important changes in the morphology and the functional heterogeneity of surrounding cells [49–51]. Our team already demonstrated the transient mesenchymal modulation of ECs by breast cancer cells inducing the constitution of an endothelial pro-tumoral niche through notch pathway [29]. This mesenchymal transformation in endothelium, called endothelial-mesenchymal transition (EndMT) [52, 53], can impact the efficacy of cancer therapy upon targeting stroma pathway [54].

The involvement of cancer derived MPs in endothelial β-catenin activation has been described for the first time in myelogenous leukemia cells [55]. The authors demonstrated that treatment with MPs derived from leukemia cells caused an increase in endothelial cell motility concomitant with a translocation of VE-cadherin and β-catenin from EC membrane [55]. More recently, human umbilical cord mesenchymal stem cells MPs were reported to induce β-catenin activation in ECs through Wnt4 leading to proangiogenic effects [56, 57].

Here, we described a mechanism explaining ovarian cancer-MPs uptake in ECs. We demonstrated that β-catenin activation was dependent on MPs internalization through αVβ3 integrins. Interestingly, αVβ3 integrins are expressed at low levels on quiescent ECs, but strongly induced on angiogenic ECs present in cancer [58]. We illustrated the increase and activation of αVβ3 integrins after OCC-MPs treatment. It has been previously described that β3 integrin antagonist could decrease MPs adhesion in ECs [59]. Additionally, Terrisse and collaborators demonstrated that MPs were internalized into ECs by interaction between the MPs surface PS and αVβ3 integrins [30]. They also illustrated that this interaction was made possible by Lactadherin. Lactadherin has already been reported to serve as a bridge between PS of MPs from apoptotic cells and integrins on phagocytic cells and plays an essential role in active endothelium in different function such as phagocytosis or uptake of apoptotic bodies [60–62]. The Lactadherin-integrin system for engulfment of apoptotic cells involves the activation of Rac1 to assemble F-actin for the formation of the phagocytic cup [63]. Interestingly, our results indicate that the uptake of OCC-MPs by ECs lead to Rac1 activation, responsible for β-catenin phosphorylation. Therefore, the interaction of OCC-MPs with ECs would lead to a release of β-catenin but also to its phosphorylation by activating Rac1/PAK1 pathway.

**Figure 6:**
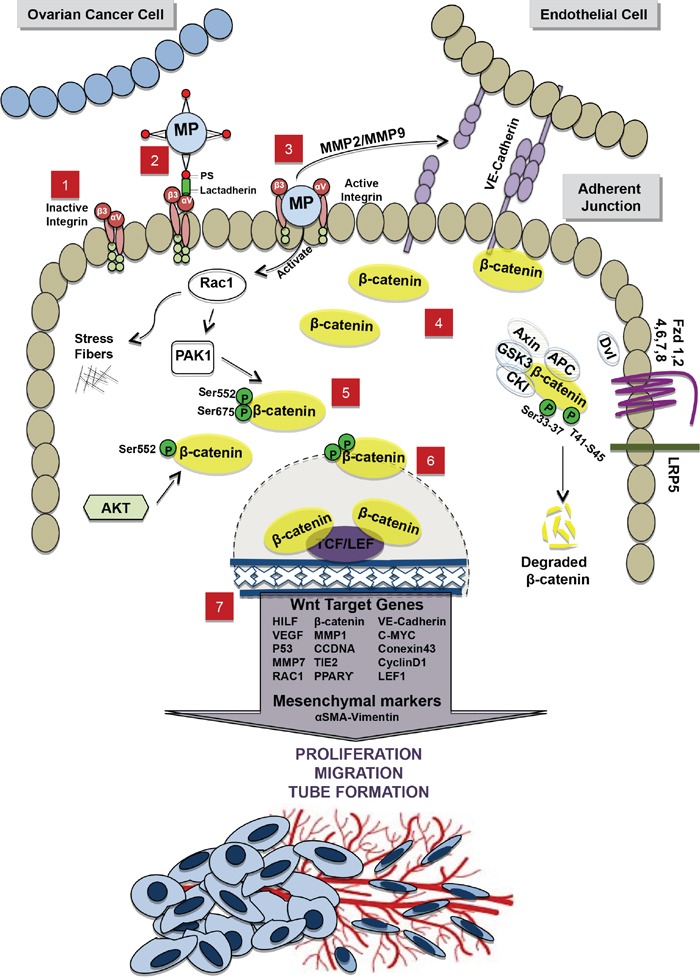
Schematic representation of the interaction between OCC-MPs and ECs **1.** In absence of OCC-MPs, integrins, αV and β3 subunits present on the EC membrane, remain inactivated **2.** OCC-MPs interact with integrins through lactadherin, expressed by EC, and phosptidylserine (PS) present on OCC-MPs surface. **3.** Internalization of OCC-MP into the EC creates 2 different activations, (i) Extracellularly, metallprotinase matrix of the OCC-MP destructs VE-Cadherin in the adherent junction and leads to the translocalization of β-catenin from the membrane to the cytoplasm. (ii) Intracellularly, OCC-MP activates Rac1, which induces polymerization of the stress fibers and the further phosphorylation of β-catenin **4.** Free β-catenin accumulates in the cytoplasm **5.** β-catenin is phosphorylated by PAK1/Rac1 pathway on 2 phosphorylation sites Ser552-Ser675 and by Akt on the phosphorylation site Ser552. **6.** The phosphorylated β-catenin is translocated to the nucleus where it interacts with the transcription factors TCF/LEF1. **7.** The interaction with TCF/LEF1 leads to the up-regulation of Wnt/β-catenin target genes and the mesenchymal markers. This activation promotes proliferation, migration and tube formation. In the off state of Wnt/β-catenin pathway, the destruction complex (APC, Axin, GSK3 and CK1) in the cytoplasm binds and phosphorylates β-catenin at 2 phosphorylation sites Ser33-37 and T41-S45. β-catenin is then ubiquinated and degraded by proteasomes.

Even if αvβ3 integrins have already been illustrated to participate to the disruption of VE-cadherin localization at ECs junctions [64–66], our data indicate that MMPs activity in OCC-MPs is also essential to MPs uptake upon integrin activation. [67]. Recent reports suggest a temporal relationship between MMPs, VE-Cadherin and β-catenin localization during angiogenic process in ECs. At an initial stage, they display more MMP-2 and MMP-9 and less VE-cadherin, while at a later stage MMPs seem to disappear and VE-cadherin and β-catenin increase at the cell junction [34]. Interestingly, MMP genes are under the transcriptional activity of β-catenin in ECs [68, 69]. It was reported that αvβ3 binds to MMP2, thereby localizing MMP-2-mediated matrix degradation to the endothelial cell surface [70]. Thus, a potential working hypothesis involves a direct physical association of MMPs from MPs and ECs, through αvβ3 integrin leading to the disruption of VE-cadherin and the release of β-catenin in the cytosol. Therefore, β-catenin happens to be free for phosphorylation by Rac1 and AKT inducing the translocation of β-catenin to the nucleus (Figure 6).

Overall our results indicate that ovarian cancer MPs could have several effects on ECs. First, loss of VE-cadherin and β-catenin at the intercellular junctions induced by the MPs could be responsible for increased motility as well as the permeable vasculature observed in solid tumor [71]. Additionally, the direct activation of β-catenin pathway by the physical association of the OCC-MPs with ECs could result in a *de novo* expression of genes involved in the neo-angiogenesis process. Altogether, our results support the fact that MPs released from cancer cells could be an important actor of angiogenesis driven by cancer cells. MPs secretion might be of clinical importance as large amounts of MPs were observed in ascites from advance-stage ovarian carcinomas [72]. A clearer understanding of the cancer cells MPs biology would provide insights into their pathophysiologic, diagnostic, and therapeutic roles.

## MATERIALS AND METHODS

### Cell cultures

Ovarian cancer cell lines Skov3 were purchased from ATCC and cultured following ATCC recommendations (ATCC, Manassas, VA, USA). A primary ovarian cancer cell line was derived in our laboratory from ascites of a patient with Stage III serous adenocarcinoma (APOCC). The cell lines were cultured in DMEM high glucose (Hyclone, Thermo Scientific), 10% FBS (Hyclone, Thermo Scientific), 1% Penicillin-Streptomycin-Amphotericyn B solution (Sigma), 1X Non-Essential Amino-Acid (Hyclone, Thermo Scientific) and 1% L-glutamine. Cultures were incubated in humidified 5% CO2 incubators at 37°C and the media was replaced every 3 days.

To avoid bias due to the use of serum, we used our model of HUVECs with autonomous Akt-activation surviving in the absence of FBS and cytokines (ECs) as a surrogate for tumor-associated endothelium [3, 29, 73–75]. E4orf1 transfected HUVEC (EC) were obtained as previously described [76]. Cells were cultured in endothelial cell growth medium (Medium 199, 20% (v/v) fetal bovine serum (FBS), 20 μg ml–1 endothelial cell growth supplement (Hallway), 1% (v/v) antibiotics (Hallway), and 20 units ml–1 heparin). Cells were serum starved 24 hours before isolation of micro-particles. In the E4+EC model the transfection of the adenoviral cassette E4orf1 in HUVECs provides low level of Akt activation allowing the use of serum-free, cytokine-free media without inducing immortalization nor altering the endothelial phenotype [76].

### Microparticles purification

MPs isolation was performed using ExoQuick™-TC (System Biosciences, Mountain View, CA) according to the manufacturer recommendations. Briefly, 48-h-supernatants of 80% serum-starved confluent tumour cells were collected, and centrifuged (4°C) at 3,000 g for 15 min to remove cells and cell debris. Supernatant aliquots of 5 mL were mixed with 1 mL of ExoQuick™-TC and incubated at 4° C overnight. The mixture was centrifuged at 1,500 g for 30 min and the MPs pellets were collected.

The final pellet containing purified MPs was either re-suspended in media for treatment of cell cultures or lysed for protein extraction or labeled for cytometry analysis or microscopy imaging. The protein concentrations of MPs were measured by Bradford assay (Biorad). Cancer cells MPs will be referred to as CC-MPs.

### Migration assay

Migration was assessed by wound closure assay as previously described [77]. Cells cultured at confluence in 24-well plates were scratched with a small tip along the ruler. Cells were then cultured for 6, 24 or 48 h in starvation media with or without MPs. The distances between the edges of the scratch were measured at 0 h and 6, 24 or 48 h after scratching. Data are represented as rate of wound closure.

### Tube formation assay

A Matrigel-based capillary-genesis assay was performed on E4+EC to assess the ability of these cells to form an organized tubular network as previously described [78]. E4+EC were starved in M199 for 6 h then 100,000 cells were cultured on 250 μl of Matrigel (BD bioscience) in tube assay medium (Medium 199, 0.2% (v/v) fetal calf serum (FCS), 10 ng ml–1 FGF2 and 20 U ml−1 heparin) with or without MPs. The degree of tube formation was quantified at different time-points by measuring the intersection of tubes in three randomly chosen fields from each well using ImageJ.

### Cell proliferation assay

Cells were plated at 50000 cells per well in a 6 well plate in medium without FBS with or without MPs. Cells were then counted with a hemocytometer for the following six days every two days. Two wells were counted per condition. For the co-culture, only the green cells (MDA-GFP) were counted. The experiment was performed in triplicate.

### Flow cytometry

Fluorescence (FL) was quantified on a SORP FACSAria2 (BD Biosciences) as previously described [75, 79]. Data were processed with FACSDiva 6.3 software (BD Biosciences). Doublets were excluded by FSC-W x FSC-H and SSC-W x SSC-H analysis. eGFP fluorescence were acquired with 488 nm blue laser and 510/50 nm emission, EpCam APC conjugated (BD Biosciences) was acquired with 647 nm red laser and 670/14 nm emission, WGA AF594 fluorescence was acquired with 535 nm green laser and 582/15 nm emission. Charts display the median of fluorescence intensity (mfi) relative to control. Single stained channels were used for compensation and fluorophore minus one (FMO) controls were used for gating. 20,000 events were acquired per sample.

### Quantification of MP uptake

To quantify MP uptake we used stained MPs (WGA AF594) and treated cell cultures for 6 or 24 h at 37°C or 4°C. Then cells were harvested and single cell suspension was analyzed by flow cytometry or fixed for confocal microscopy imaging.

### Confocal microscopy

ECs treated with MPs as detailed in specific experiments were fixed in 3.7% formaldehyde. Slides were mounted in a mounting media SlowFade® Gold Antifade Reagent (Invitrogen). Fluorescence Imaging was performed using a Zeiss confocal Laser Scanning Microscope 710 (Carl Zeiss). Post-acquisition image analysis was performed with Zeiss LSM Image Browser Version 4.2.0.121 (Carl Zeiss).

### Imagexpress screening microscopy

The ImageXpress platform (Molecular Devices LLC, Sunnyvale, CA) is a widefield automated microscope capable of fluorescent, transmitted light, and phase-contrast imaging of fixed- or live-cells. The system optical drive includes a 300-W Xenon lamp as broad-spectrum white light source and 2/3 chip cooled CCD camera and optical train for standard field of view imaging and a transmitted light option with phase contrast. After incubating endothelial cells with CCs-MPs (from 15 minutes to 24h), cells were stained for beta catenin or Ve-Cadherin. 100 pictures were taken per wells (4 wells per condition) and images were analysed with MetaXpress® High-Content Image Analysis Software (Molecular Devices LLC, Sunnyvale, CA).

### Western blot analysis

Western blot was carried out as previously described [80]. Immunostaining was carried out using a goat monoclonal antibodies against Integrins: α1, α5, α6, αV and β3 (#ab34445, #ab150361, #ab20142, #ab179475, #ab75872, abcam) and Wnt/β-Catenin Activated Targets Antibody Sampler Kit (Cell Signaling # 8655S), β-Catenin Antibody Sampler Kit (Cell Signaling #2951S), Rho-GTPase Antibody Sampler Kit(Cell Signaling, #9968S), actin (1/1000, Cell signaling) and a secondary polyclonal mouse anti-goat antibody HRP conjugated (1/2000, cell signaling). Blots were developed using HRP and chemiluminescent peroxidase substrate (#CPS1120, Sigma). Data were collected using Geliance CCD camera (Perkin Elmer), and analyzed using ImageJ software (NIH).

### Zymogram

The activity of MMPs inside the OCC-MPs was determined by gelatin zymography, 10% polyacrylamide gel polymerized together with gelatin (1 mg/ml) (Novex by life technologies Lot no.:14051365).

Cells are lysed using 2x tricine SDS sample buffer (3M Tris HCL pH8.45, 12%glycerol, 4%SDS, 0.1% commassie blue, 0.1% phenol red). After electrophoresis, gels were placed in renaturing buffer (Novex by life technologies, Lot no.:1570233) for 30mins at RT and then in developing buffer (Novex by life technologies, Lot no.:1563634) for 30 minutes at RT. After an overnight incubation at 37°C in developing buffer, gels are stained with blue stain (Invitrogen Simply blue Safestain TM Cat no.: LC6060) for 1h at RT. After 1 to 2h of destaining process, gels are finally developed, and the Gelatinolytic activities appearing as clear zone were quantitated.

### RT-PCR analysis

Total RNA was extracted from cell cultures using Trizol. After genomic DNA removal by DNase digestion (Turbo DNA free kit, Applied Biosystems), total RNA (1μg) was reverse transcribed with oligodT (Promega) using the Superscript III First-Strand Synthesis SuperMix (Invitrogen). PCR analysis was performed as previously described [3] with a MasterCycler apparatus (Eppendorf) from 2 μL of cDNA using primers from IDT (Table 1).

**Table 1:** List of primers

Primer Name	Forward	Reverse
C-myc	TGCTCCATGAGGAGACACCG	ATGTGTGGAGACGTGGCACC
CCDN1	GTCCATGCGGAAGATCGTCG	TCTCCTTCATCTTAGAGGCCACG
Connexin43	AACTGCTGGAGGGAAGGTGTGG	CATGAGCCAGGTACAAGAGTGTGG
Cyclin-D1	CCCGCACGATTTCATTGAAC	GCGGAT TGGAAATGAACTTCAC
Dkk1	CAGGATTGTGTTGTGCTAGA	TGACAAGTGTGAAGCCTAGA
Dkk2	CTCAACTCCATCAAGTCCTC	TACCTCCCAACTTCACACTC
Dkk3	GAGGTTGAGGAACTGATGG	CCAGTCTGGTTGTTGGTTAT
Dkk4	GTCCTGGACTTCAACAACAT	GTTGCATCTTCCATCGTAGT
Fzd1	GAACTTTCCTCCAACTTCATGGG	CATTTCCATTTTACAGACCGG
Fzd10	ACACGTCCAACGCCAGCATG	ACGAGTCATGTTGTAGCCGATG
Fzd2	GGTGAGCCAGCACTGCAAGAG	CCTAAAAGTGAAATGGTTTCGATCG
Fzd3	GCTGTACTCACAGTTAACATG	GCTAAAATACCCTTGCTAGATTT
Fzd4	TGCCTTTTCAGGGCAAAGTG	ACAGGAAGAGATTTATGGAATG
Fzd5	TACCCAGCCTGTCGCTAAAC	AAAACCGTCCAAAGATAAACTGC
Fzd6	TGGCCTGAGGAGCTTGAATGTGAC	TATCGCCCAGCAAAAATCCAATGA
Fzd7	GTTTGGATGAAAAGATTTCAGGC	GACCACTGCTTGACAAGCACAC
Fzd8	ACAGTGTTGATTGCTATTAGCATG	GTGAAATCTGTGTATCTGACTGC
Fzd9	CCCTAGAGACAGCTGACTAGCAG	CGGGGGTTTATTCCAGTCACAGC
GADPH	ACATCAGCCAAGTCAATGTTTCG	AGCATTAACAGCAACAATCCGG
HIF1a	TCGGCGAAGTAAAGAATCTGAA	CAAATCACCAGCATCCAGAAG
LEF1	AGAGAAAGGAGCAGGAGCCAAA	ACACTCAGCAACGACATTCGC
LRP5	GACATCTACAGCCGGACACTG	CACAAGTCAGCAGGTTCTGCAGG
LRP5	GACATCTACAGCCGGACACTG	CACAAGTCAGCAGGTTCTGCAGG
LRP6	GATTATCCAGAAGGCATGGCAG	CAATCACCATGCGGTTGATGGC
LRP6	GATTATCCAGAAGGCATGGCAG	CAATCACCATGCGGTTGATGGC
MMP1	GCC TGA TGT GGC TCA GTT TGT CC	TGT CTG CTT GAC CCT CAG AGA CC
MMP7	TGTATGGGGAACTGCTGACA	GCGTTCATCCTCATCGAAGT
P53	CGT CAGAAGCACCCAGGACT	CAT CCTCCTCCCCACAAC AA
PPARγ	AGT GGC TCA GGA CTC TCT	TGG CCG CAG AAA TGA CCA
Rac1	ATG CAG GCC ATC AAG TGT GTG GTG	TTA CAA CAG CAG GCA TTT TCT CTT CC
SFRP1	GGACCGGCCCATCTACCC	TCCTTGTTTTTCTTGTCCCACTTG
SFRP2	CTCGCTGCTGCTGCTCTTC	GGCTTCACATACCTTTGGAG
SFRP3	ATGGTCTGCGGCAGCCCGG	CTGTCGTACACTGGCAGCTC
SFRP4	GTTCCTCTCCATCCTAGTGG	GCTGAGATACGTTGCCAAAG
SFRP5	CTACTGGAGGGTGTTTTCAC	CTTTCCCTTACCCTCTCCT
TCF1	TCAATCTGCTCATGCATTACC	AGGTCAGGGAGTAGAAGCCAG
TIE2	TTA TTT CTG TGA AGG GCG AGT TCG	AAT ATC AGG TAC TTC ATG CCG GG
VE-Cadherin	GAAGCCTCTGATTGGCACAGTG	TTTTGTGACTCGGAAGAACTGGC
VEGF	CGCCTCTCCAAAAAGCTACAC	CTCACAGGAAACCGGACATC
WIF-1	CACCTGGATTCTATGGAGTG	ACAGAGGTCTCCCTGGTAAC
Wnt1	CAACAGCAGTGGCCGATGGTGG	CGGCCTGCCTCGTTGTTGTGAAG
Wnt10a	CTGTTCTTCCTACTGCTGCT	ACACACACCTCCATCTGC
Wnt10b	GCACCACAGCGCCATCCTCAAG	GGGGTCTCGCTCACAGAAGTCAGGA
Wnt11	CTGGAAATGAAGTGTAAGTGC	TGTGTCCCGTGGGAGCCCACC
Wnt14	ACAAGTATGAGACGGCACTC	AGAAGCTAGGCGAGTCATC
Wnt15	TGAAACTGCGCTATGACTC	GTGAGTCCTCCATGTACACC
Wnt16	GAGAGATGGAACTGCATGAT	GATGGGGAAATCTAGGAACT
Wnt2	GTCATGAACCAGGATGGCACA	TGTGTGCACATCCAGAGCTTC
Wnt2b	AAGATGGTGCCAACTTCACCG	CTGCCTTCTTGGGGGCTTTGC
Wnt3	GAGAGCCTCCCCGTCCACAG	CTGCCAGGAGTGTATTCGCATC
Wnt3a	CAGGAACTACGTGGAGATCATG	CCATCCCACCAAACTCGATGTC
Wnt4	TGCCACTGAGGTGGAGCCAC	TCAGCCAGCTCCACCTGCGC
Wnt5a	GACCTGGTCTACATCGACCCC	GCAGCACCAGTGGAACTTGCA
Wnt5b	TGAAGGAGAAGTACGACAGC	CTCTTGAACTGGTTGTAGCC
Wnt6	TTATGGACCCTACCAGCAT	ATGTCCTGTTGCAGGATG
Wnt7a	GCCGTTCACGTGGAGCCTGTGCGTGC	AGCATCCTGCCAGGGAGCCCGCAGCT
Wnt7b	GATTCGGCCGCTGGAACTGCTC	TGGCCCACCTCGCGGAACTTAG
Wnt8a	CTGGTCAGTGAACAATTTCC	GTAGCACTTCTCAGCCTGTT
Wnt8b	GTCTTTTCACCTGTGTCCTC	AGGCTGCAGTTTCTAGTCAG
Β-catenin	GCTGCTGTTTTGTTCCGAATG T	GCCATTGGCTCTGTTCTGAAGA

### Statistical analysis

All quantitative data were expressed as mean ± standard error of the mean (SEM). Statistical analysis was performed with SigmaPlot 11 (Systat Software Inc., Chicago, IL). A Shapiro-Wilk normality test, with a p = 0.05 rejection value, was used to test normal distribution of data prior further analysis. All pairwise multiple comparisons were performed by one way ANOVA followed by Holm-Sidak posthoc tests for data with normal distribution or by Kruskal-Wallis analysis of variance on ranks followed by Tukey posthoc tests, in case of failed normality test. Paired comparisons were performed by Student's t-tests or by Mann-Whitney rank sum tests in case of unequal variance or failed normality test. Statistical significance was accepted for p < 0.05 (*), p < 0.01 (**) or p < 0.001 (***). All experiments were performed in triplicates.

## SUPPLEMENTARY FIGURES


