# Supplementary material for: VE-cadherin cleavage by ovarian cancer microparticles induces β-catenin phosphorylation in endothelial cells
Source: Oncotarget. 2015 Dec 19;7(5):5289–305. doi: 10.18632/oncotarget.6677 (PMC4868686; doi:10.18632/oncotarget.6677)
Supplement: Supplementary file 1 [file oncotarget-07-5289-s001.pdf]

SUPPLEMENTARY FIGURES

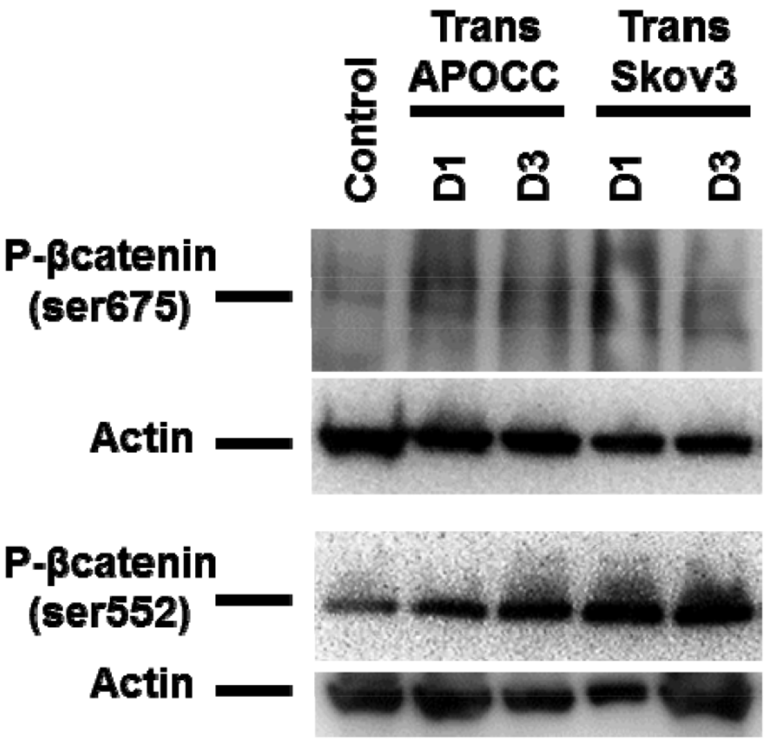

Supplementary Figure S1: Transwell experiment. The co-culture of E4+ECs and OCC was repeated using transwell chamber. Western blot reveals a phosphorylation of β-catenin for the sites studied after co-culture with Skov3 or APOCC. $p < 0.05$ (*), $p < 0.01$ (**) or $p < 0.001$ (***).

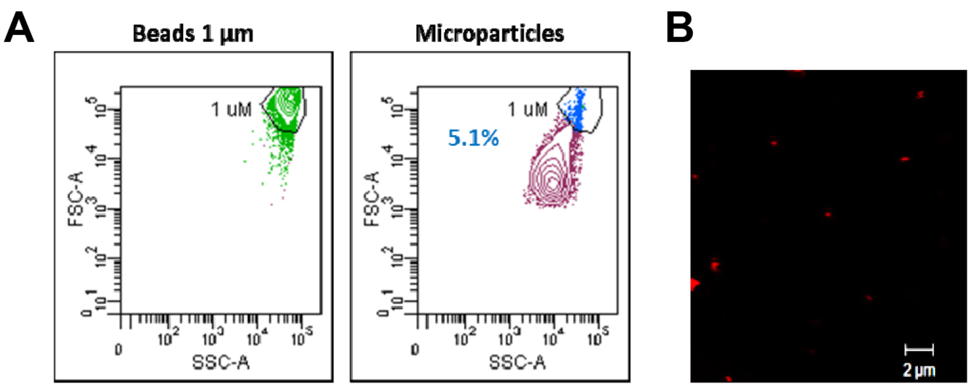

Supplementary Figure S2: A. Characterization of OCC-MPs by flow cytometry. 1 µm beads were used to calibrate the gate. OCC-MPs are smaller than 1 µm **B.** Characterization of OCC-MPs by confocal microscopy. OCC-MPs are smaller than 1 µm.

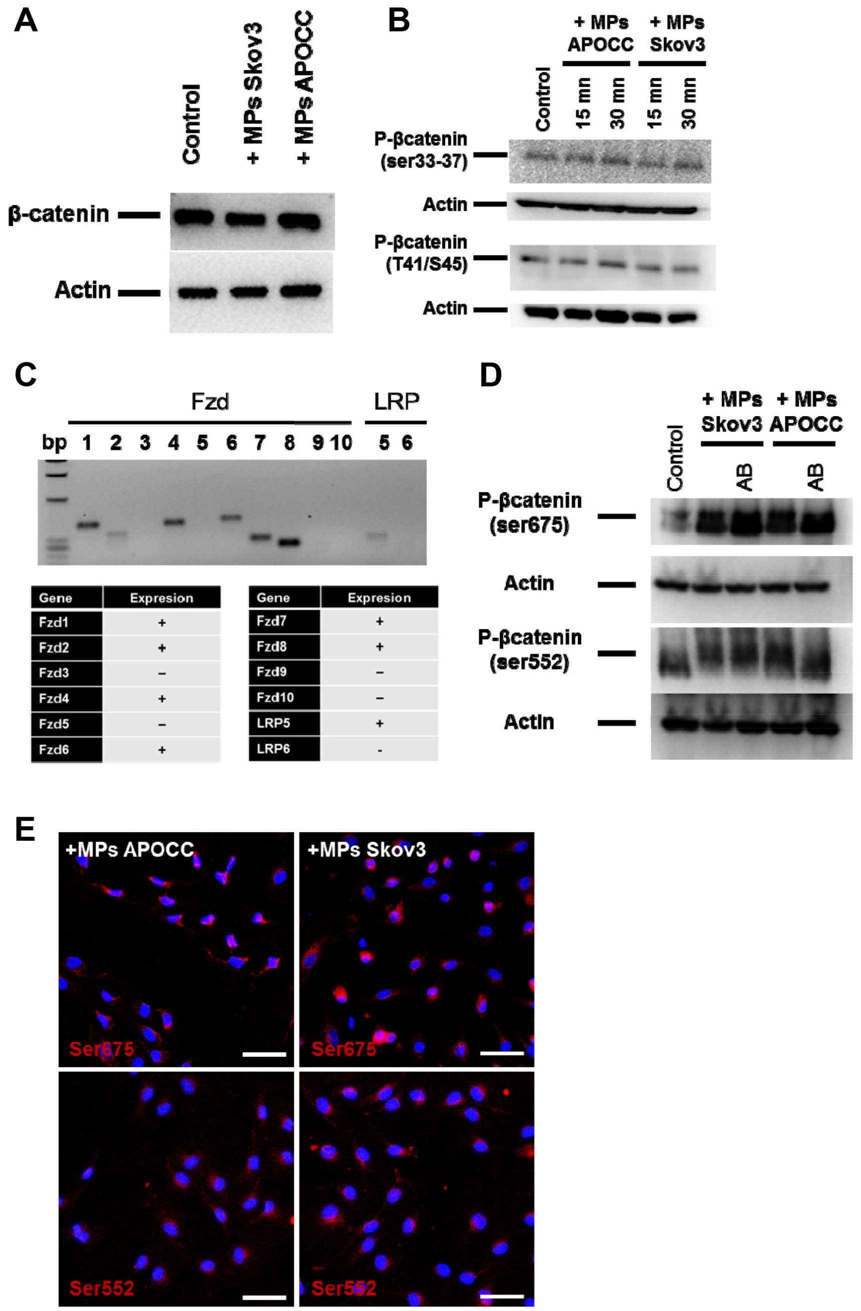

Gene	Expresion
Fzd1	+
Fzd2	+
Fzd3	–
Fzd4	+
Fzd5	–
Fzd6	+

Gene	Expresion
Fzd7	+
Fzd8	+
Fzd9	–
Fzd10	–
LRP5	+
LRP6	-

Supplementary Figure S3: A. Quantification of β-catenin. E4+ECs were serum starved for 24 hours and treated or not (control) with MPs from Skov3 or APOCC for 30 minutes. No increase of β-catenin could be observed in the treated samples. **B.** Quantification of β-catenin phosphorylation at Ser33–37 and T41-S45. E4+ECs were serum starved for 24 hours and treated or not (control) with MPs from Skov3 or APOCC for 15 or 30 minutes. No increase of β-catenin phosphorylation on the two sited studied could be observed in the treated samples. **C.** Frizzled and LRP expression in E4+ECs. PCR for all human primers for frizzled 1 to 10 and LRP5 and 6 have been performed (top panel). The table summarizes the Frizzled and LRP expression by our cells. **D.** β-catenin phosphorylation in the presence of frizzled inhibitor. E4+ECs, serum-starved for 24 h, were treated or not with a cocktail of antibodies against frizzled 1, 2, 4, 6, 7, 8 and LRP5 6 hours prior the incubation with OCC-MPs during 15 minutes. Western blots for the phosphorylation of β-catenin at the sites Ser675 and Ser552 were performed. The inhibition of frizzled and LRP didn't modify the phosphorylation of β-catenin induced by OCC-MPs. **E.** Phospho-β-catenin localization. E4+ECs, serum-starved for 24 h, were treated with a cocktail of antibodies against frizzled 1, 2, 4, 6, 7, 8 and LRP5 6 hours prior the incubation with OCC-MPs during 1 hour. E4+ECs were stained for the phosphorylation of β-catenin at the sites Ser675 and Ser552 and analysed by confocal microscopy. The inhibition of frizzled and LRP didn't modify the phosphorylation of β-catenin and its translocation induced by OCC-MPs.

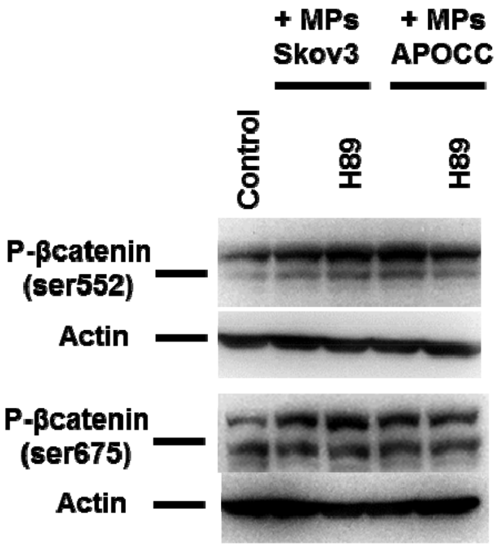

Supplementary Figure S4: Phosphorylation of β-catenin in the presence of PKA inhibitor. E4+ECs, serum-starved for 24 h, were pre-treated or not with H-89 (10 μM). Phosphorylation of β-catenin at the sites Ser675 and Ser552 were analyzed by western blot. H-89 was not able to reduce the phosphorylation of β-catenin at Ser552 neither at Ser675.
